# Relationship of circulating total bilirubin, UDP-glucuronosyltransferases 1A1 and the development of non-alcoholic fatty liver disease: a cross-sectional study

**DOI:** 10.1186/s12876-021-02088-7

**Published:** 2022-01-05

**Authors:** Xuefeng Ma, Xu Zheng, Shousheng Liu, Likun Zhuang, Mengke Wang, Yifen Wang, Yongning Xin, Shiying Xuan

**Affiliations:** 1 Department of Infectious Disease, Qingdao Municipal Hospital, Qingdao University, 1 Jiaozhou Road, Qingdao, 266011 Shandong Province China; 2 Department of Laboratory Medicine, Qingdao Municipal Hospital, Qingdao University, Qingdao, 266011 China; 3 Clinical Research Center, Qingdao Municipal Hospital, Qingdao University, Qingdao, 266071 China; 4 Digestive Disease Key Laboratory of Qingdao, Qingdao, 266071 China

**Keywords:** Nonalcoholic fatty liver disease, UDP-glucuronosyltransferase 1A1, Bilirubin, Magnetic resonance imaging proton density fat fraction

## Abstract

**Background:**

This study aimed to investigate the correlation of circulating total bilirubin (TB) and UGT1A1 with NAFLD in Chinese Han population.

**Methods:**

172 adults were enrolled from the Qingdao Municipal Hospital from May 2019 to October 2020. All individuals were examined with MRI-PDFF and divided into no steatosis, mild steatosis, moderate steatosis, and severe steatosis groups according to the MRI-PDFF values. The biochemical indexes and UGT1A1 were measured.

**Results:**

There was no significant difference of circulating TB and UGT1A1 levels between NAFLD group and controls. In the moderate steatosis and severe steatosis groups, the circulating TB levels were higher than that in control group (all *P* < 0.05). In addition, circulating TB levels were weak positively associated with liver fat fraction in NAFLD patients (ρ = 0.205, *P* = 0.001). There was no significant correlation between circulating UGT1A1 levels with liver fat fraction in patients with NAFLD (ρ = 0.080, *P* = 0.179), but positively correlation was found in patients with severe steatosis (ρ = 0.305, *P* = 0.026).

**Conclusions:**

The circulating TB levels were significant high in patients with moderate and severe steatosis. Circulating TB levels were weakly associated with liver fat fraction in patients with NAFLD, and the circulating UGT1A1 levels were positively correlated with liver fat fraction in NAFLD patients with severe steatosis.

*Trial registration*: ChiCTR, ChiCTR1900022744. Registered 24 April 2019 – Retrospectively registered, http://www.chictr.org.cn/edit.aspx?pid=38304&htm=4.

## Background

Nonalcoholic fatty liver disease (NAFLD) was the most prevalent chronic liver disease with a mean prevalence of 20–30% in the world [1, 2]. NAFLD is a clinicopathological disorder whose spectrum ranges from benign steatosis to nonalcoholic steatohepatitis (NASH) [3]. Although liver biopsy is recognized as the golden method for the diagnosis of NASH, the invasive feature limits its clinical application in the routine health examination. Magnetic resonance imaging proton density fat fraction (MRI-PDFF) has been proven to has a better correlation with histology-proven steatosis grade and becomes the important method for steatosis quantification with the high sensitivity, specificity, and reliability [4].

Bilirubin is the by-product of hemoglobin catabolism that released from the red blood cells and possesses the antioxidant and cytoprotective function [5, 6]. Low concentration of bilirubin is associated with the benefical antioxidant acitivities and high circulating bilirubin level is associated serious toxicities [7]. The excretion of bilirubin in vivo is controlled by the UDP-glucuronosyltransferase 1A1 (UGT1A1), which could mediate the glucuronidation of bilirubin to make it more soluble for biliary excretion [8]. Prior to this, some studies had investigated the role of circulating bilirubin in NAFLD. Chang et al. investigated the relationship between circulating bilirubin levels and the developing risk of NAFLD by a large prospective study, and they found that circulating direct bilirubin were negatively associated with the developing risk of NAFLD, while NAFLD patients usually had the low levels of circulating direct bilirubin [9]. Same as in the children, Kanika et al. found that circulating bilirubin levels were inverse related to the risk of NASH that diagnosed by biopsy [10]. However, Tian et al. reported that direct bilirubin was negatively related to the developing risk of NAFLD, the circulating levels of indirect bilirubin and TB were not associated with the developing risk of NAFLD [11]. Kunutsor et al. also found that TB was not causally associated with the risk of MR-diagnosed NAFLD [12].

UGT1A1 plays an important role in the secretion of bilirubin, and the activity of UGTA1A protein is influenced by the genetic and environmental factors [7]. Several studies had explored the association between UGT1A1 gene variants (UGT1A1 ∗ 6 and UGT1A1 ∗ 28) and the risk of developing NAFLD. Luo et al. analyzed the variants of UGT1A1 and suggested that bilirubin could induced the development of NAFLD causally [13]. But the research conducted by Lin et al. demonstrated that variant UGT1A1*6 genotypes possessed the protective effect on the risk of NAFLD in obese Taiwanese children [14]. So far, no study reported the correlation between circulating UGT1A1 levels and the developing risk of NAFLD. In consideration of the inconsistent conclusion of circulating TB and the absent data of circulating UGT1A1 with the risk of NAFLD, this study was designed to investigate the correlation between circulating TB and UGT1A1 levels with the MRI-PDFF diagnosed NAFLD in Chinese Han population.

## Methods

### Research subjects

From May 2019 to October 2020, 172 subjects were enrolled from Qingdao Municipal Hospital. This study was approved by the Ethics Committee of the Qingdao Municipal Hospital and all participants signed the informed consent. Inclusion criteria for patients with NAFLD were as follows: (1) 18–65 years of age; (2) NAFLD was diagnosed by MRI-PDFF; (3) Patients without the infection by hepatitis B virus, hepatitis C virus, or other bacteria and viruses; (4) No other liver diseases or tumors; (5) No drinking or ≤ 70 g ethanol/week for women (about one standard drink daily) and ≤ 140 g ethanol/week for men (two standard drinks daily); (6) No history of medicine in recent 1 year; (7) No pregnancy for women. If the MRI-PDFF values were more than 5%, they will be distributed to NAFLD group, otherwise, they will be distributed to healthy control group if they were under the healthy condition.

### Data collection

The information of age, gender, history of medicine and smoking, and diet were obtained by the standard questionnaire. The physical indexes such as height, weight, waistline hip circumference were measured by the standard measurement method. Body mass index (BMI) was calculated as the equation of body weight/height^2^. Blood sample of each subject was taken after a 12-h overnight fasting. The biochemical indexes such as serum TB, alanine aminotransferase (ALT), total cholesterol (TC), triglyceride (TG), aspartate aminotransferase (AST), γ-glutamyltransferase (GGT), alkaline phosphatase (ALP), fasting plasma glucose (FPG), low-density lipoprotein (LDL), high-density lipoprotein (HDL), uric acid (UA), apolipoprotein A1 (APOA1), apolipoprotein B (APOB), and insulin, which were measured by using a Beckman Clinical Chemistry System (Beckman, California, USA). Glycated hemoglobin (HbA1c) was measured by Tosoh Automated glycohemoglobin analyzer (Tosoh, Yamaguchi, Japan), and the UGT1A1 was measured by ELISA detection kit (Shanghai Enzyme-linked Biotechnology, Shanghai, China).

### MRI-PDFF examination

All individuals underwent an abdominal MRI-PDFF examination for the diagnosis of NAFLD using a 3.0 T MRI Ingenia system (Philips Healthcare, Best, the Netherlands) according to the methods of Zhang et al. [15]. Participants with a MRI-PDFF value ≥ 5% was diagnosed as the NAFLD patients. The mild steatosis (S0) was defined as 5% ≤ MRI < 16.3%, the moderate steatosis (S1) was defined as 16.3% ≤ MRI-PDFF < 21.7%, the severe steatosis (S2) was defined as MRI-PDFF ≥ 21.7% [4, 16].

### Statistical analysis

Continuous variables were presented as mean ± standard deviation (SD) and categorical variables were expressed as frequencies or percentages. The qualitative and quantitative difference between subgroups was analyzed by chi-square test for categorical parameters, and continuous parameters were analyzed by Mann–Whitney’s test or Student’s t-test. The correlations of liver fat fraction with the clinical and biochemical parameters were compared based on the Spearman rank correlation coefficient. The correlation of liver fat fraction with TB or UGT1A1 were classified as negligible (ρ < 0.10), weak (0.10 < ρ < 0.39), moderate (0.40 < ρ < 0.69), strong (0.70 < ρ < 0.89) and very strong (ρ > 0.90). The odds ratio (OR) and its 95% confidence interval (95% CI) were calculated using multivariable analysis to investigate the impact of circulating UGT1A1 on the developing risk of NAFLD. Statistical analysis was conducted used SPSS 26.0 (IBM, USA) and *P* < 0.05 was considered as the statistical difference.

## Results

### Demographic characteristics

Clinical characteristics of participants were showed in the Table 1. The included subjects comprise of 125 patients with NAFLD (mean age 42.73 ± 13.03 years) and 47 healthy controls (mean age 42.81 ± 12.49). No significant difference of age in each group was found (all *P* > 0.05). According to the MRI-PDFF values, NAFLD patient were divided into three subgroups: mild (n = 79), moderate (n = 20), and severe (n = 26) steatosis group. The mean BMI value and serum levels of UA, ALT, TC, LDL, and insulin in NAFLD patients were higher than that in control group (all *P* < 0.05), and the serum HDL level was lower in NAFLD patients than that in controls (*P* < 0.05). In the mild steatosis group, the BMI value and serum levels of ALT, ALP, UA, TC, APOB, and insulin were higher than that in control group (all *P* < 0.05). In the moderate group, the BMI value and serum levels of TC, AST, ALT, APOB, insulin, and TB were higher than in controls (all *P* < 0.05). The BMI value and serum levels of AST, ALT, UA, TC, insulin, and TB in severe steatosis group were higher than in healthy controls (all *P* < 0.05), and the serum HDL levels in patients with severe steatosis were lower than that in controls (*P* < 0.05). Although no statistical difference was existed, the serum LDL levels in mild steatosis group, moderate steatosis group, and severe steatosis group were all higher than in controls.

**Table 1:** Characteristics of the Subjects that stratified according to MRI-PDFF value

	Controls (n = 47)	NAFLD (n = 125)	Mild (n = 79)	Moderate (n = 20)	Severe (n = 26)
Gender (M/F)	16/31	83/42	51/28	12/8	20/6
Age (years)	42.81 ± 12.49	42.73 ± 13.03	44.51 ± 13.43	41.50 ± 11.39	37.50 ± 11.73
BMI (kg/m^2^)	24.67 ± 3.99	27.98 ± 3.88^a^	27.03 ± 3.44^b^	28.52 ± 3.76^c^	30.64 ± 4.11^d^
TB (μmol/L)	13.42 ± 4.31	15.65 ± 7.84	15.06 ± 8.82	16.51 ± 5.96^c^	16.81 ± 5.55^d^
ALT (U/L)	30.33 ± 32.35	55.84 ± 42.85 ^a^	43.86 ± 37.93^b^	58.77 ± 35.19^c^	90.88 ± 44.53^d^
AST (U/L)	26.43 ± 17.44	34.81 ± 20.80	31.03 ± 22.24	35.37 ± 14.87^c^	46.16 ± 16.01^d^
ALP (U/L)	77.04 ± 28.73	87.88 ± 26.49	91.27 ± 24.34^b^	81.30 ± 20.82	83.39 ± 34.71
GGT (U/L)	38.52 ± 56.12	54.52 ± 52.17	52.01 ± 53.39	51.28 ± 44.51	64.44 ± 54.96
FPG (mmol/L)	5.05 ± 1.35	5.73 ± 1.95	5.87 ± 2.31	5.64 ± 1.06	5.38 ± 0.98
UA (μmol/L)	362.10 ± 115.67	427.20 ± 122.89^a^	406.06 ± 115.54^b^	410.21 ± 105.99	503.37 ± 130.70^d^
TC (mmol/L)	1.52 ± 1.38	2.77 ± 2.55^a^	2.48 ± 2.79^b^	3.34 ± 2.05^c^	3.18 ± 2.02^d^
TG (mmol/L)	4.72 ± 1.42	4.93 ± 1.59	5.06 ± 1.61	4.51 ± 1.67	4.89 ± 1.46
LDL (mmol/L)	2.63 ± 0.88	2.96 ± 0.92 ^a^	2.93 ± 0.83	2.98 ± 2.63	3.07 ± 1.33
HDL (mmol/L)	1.50 ± 0.52	1.29 ± 0.56^a^	1.32 ± 0.65	1.26 ± 0.40	1.23 ± 0.36^d^
APOA1 (g/L)	1.45 ± 0.54	1.57 ± 2.56	1.72 ± 3.20	1.37 ± 0.34	1.28 ± 0.54
APOB (g/L)	1.02 ± 0.32	1.15 ± 0.30	1.15 ± 0.34^b^	1.19 ± 0.25^c^	1.13 ± 0.22
Insulin (%)	7.98 ± 4.05	14.96 ± 15.11^a^	14.46 ± 18.22^b^	13.94 ± 5.66^c^	17.46 ± 5.98^d^
HbA1c (μmol/L)	5.61 ± 0.90	5.99 ± 1.38	6.02 ± 1.57	5.94 ± 0.65	5.92 ± 1.15
UGT1A1 (ng/mL)	1.63 ± 1.59	1.72 ± 1.56	1.64 ± 1.38	1.96 ± 1.82	1.74 ± 1.91
MRI-PDFF (%)	3.92 ± 1.15	16.30 ± 7.93^a^	11.25 ± 3.33^b^	19.09 ± 2.42^c^	27.86 ± 5.68^d^

BMI, body mass index; TB, total bilirubin; ALT, alanine aminotransferase; AST, aspartate aminotransferase; ALP, alkaline phosphatase; GGT, γ-glutamyltransferase; FPG, fasting plasma glucose; UA, uric acid; TC, total cholesterol; TG, triglyceride; LDL, low-density lipoprotein; HDL, high-density lipoprotein; APOA1, apolipoprotein A1; APOB, apol ipoprotein B; HbA1c, Glycated hemoglobin; UGT1A1, UDP-glucuronosyltransferase 1A1

^a^NAFLD versus controls, *P* < 0.05

^b^Mild steatosis versus controls, *P* < 0.05

^c^Moderate steatosis versus controls *P* < 0.05

^d^Severe steatosis versus controls, *P* < 0.05

### Correlation between the circulating TB and UGT1A1 levels

To explore the correlation of the circulating TB and UGT1A1 levels, Spearman rank correlation coefficient analysis were conducted. There was no correction between the circulating TB and UGT1A1 levels in NAFLD patients (ρ = 0.023, *P* = 0.767) and all subjects (ρ = 0.081, *P* = 0.383).

### Correlation between liver fat fraction and circulating TB

To explore the correlation of circulating TB with the liver fat fraction, Spearman rank correlation coefficient analysis was conducted. As shown in the Table 2, the circulating level of TB was weak positively associated with liver fat fraction which presented by the MRI-PDFF values in NAFLD patients (ρ = 0.259, *P* = 0.006) and all subjects (ρ = 0.214, *P* = 0.005). In addition, circulating UGT1A1 levels were divided into quartiles. The quartile 1 included subjects with circulating UGT1A1 levels ≤ 1.02 ng/mL, quartile 2 was 1.03–1.33 ng/mL, quartile 3 was 1.34–1.73 ng/mL, and quartile 4 was ≥ 1.74 ng/mL. The correlation of circulating TB with the liver fat fraction in each quartile was shown in the Table 3. Multivariable analysis was conducted to investigate the impact of circulating TB on the developing risk of NAFLD. As the results shown in the Table 5, the circulating TB could increase the developing risk of NAFLD (OR 1.136, 95% CI 1.069–1.208, *P* < 0.001). After adjusted to the age, sex, and BMI [17–19], no significant association of circulating TB with the developing risk of NAFLD (OR 1.028, 95% CI 0.954–1.108, *P* = 0.471) was found.

**Table 2:** Correlation between liver fat fraction with the circulating TB and UGT1A1 in NAFLD patients and all subjects

	Spearman correlation coefficient	*P*
NAFLD		
TB	0.259	0.006
UGT1A1	0.080	0.179
All subjects		
TB	0.214	0.005
UGT1A1	0.088	0.256

**Table 3:** Correlation between liver fat fraction with the circulating TB among each quartile

	Spearman correlation coefficient	*P*
Quartile 1	0.078	0.620
Quartile 2	0.186	0.227
Quartile 3	0.230	0.137
Quartile 4	0.204	0.190

### Correlation between liver fat fraction and circulating UGT1A1

To explore the correlation of circulating UGT1A1 with the liver fat fraction, Spearman rank correlation coefficient analysis were conducted. As shown in the Table 2, no significant correlation was found between the circulating level of UGT1A1 with liver fat fraction in NAFLD patients (ρ = 0.080, *P* = 0.179) and all subjects (ρ = 0.088, *P* = 0.256). To investigate the potential influence factor to the correlation, the relationship of circulating level of UGT1A1 with liver fat fraction were analyzed in patients with mild steatosis, moderate steatosis, and severe steatosis, respectively. The results suggested that there was no obvious correlation of circulating level of UGT1A1 with liver fat fraction in patients with mild steatosis and moderate steatosis, but circulating UGT1A1 levels were positively associated with liver fat fraction in patients with severe steatosis (ρ = 0.305, *P* = 0.026) (Table 4).

**Table 4:** Correlation between circulating UGT1A1 levels with liver fat fraction in patients with different grades of steatosis

	Spearman correlation coefficient	*P*
Mild steatosis	0.1	0.391
Moderate steatosis	− 0.205	0.221
Severe steatosis	0.305	0.026

Multivariable analysis was conducted to investigate the impact of circulating UGT1A1 on the developing risk of NAFLD. As the results shown in the Table 5, the circulating UGT1A1 was not associated with the developing risk of NAFLD (OR 0.994, 95% CI 0.905–1.091, *P* = 0.19).

**Table 5:** Logistic analysis of the impact of TB and UGT1A1 on the risk of NAFLD

	OR (95% CIs)	*P*	Adjusted^a^ OR (95% CIs)	*P*
TB	1.136 (1.069–1.208)	< 0.001	1.028 (0.954–1.108)	0.471
UGT1A1	0.994 (0.905–1.091)	0.19		

CI, confidence interval; OR, odds ratio

^a^Model adjusted for age, sex, and BMI

### Correlation between liver fat fraction with circulating UGT1A1 and TB in groups stratified by TB, ALT, and gender

In order to investigate the possible relationship between liver fat fraction with circulating TB and UGT1A1, NAFLD patients were stratified by gender and the levels of TB and ALT. As the results showed in Table 6, there was no significant correlation of circulating UGT1A1 with liver fat fraction (ρ = 0.400, *P* = 0.327; ρ = 0.032, *P* = 0.545, respectively) in both the high TB group (TB > 17.1) [20] and normal TB group (TB ≤ 17.1). Both in the high ALT group (ALT > 45) and normal ALT group (ALT ≤ 45) [21], no obvious correlations of circulating UGT1A1 and TB with liver fat fraction were observed (ρ = 0.186, *P* = 0.178; ρ = − 0.016, *P* = 0.908; ρ = 0.022, *P* = 0.816; ρ = 0.069, *P* = 0.095, respectively). Same to in the male patients and female patients, there were also no significant correlation between circulating UGT1A1 and TB with liver fat fraction (ρ = 0.145, *P* = 0.158; ρ = 0.099, *P* = 0.341; ρ = − 0.049, *P* = 0.683; ρ = 0.166, *P* = 0.167, respectively) (Table 6).

**Table 6:** Correlation between liver fat fraction with circulating UGT1A1 and TB in groups stratified by TB, ALT, and gender

	Spearman correlation coefficient	*P*
High TB		
UGT1A1	0.400	0.327
Normal TB		
UGT1A1	0.032	0.545
High ALT		
UGT1A1	0.186	0.178
TB	− 0.016	0.908
Normal ALT		
UGT1A1	0.022	0.816
TB	0.069	0.095
Male		
UGT1A1	0.145	0.158
TB	0.099	0.341
Female		
UGT1A1	− 0.049	0.683
TB	0.166	0.167

## Discussion

Bilirubin is the by-product of hemoglobin catabolism and possesses the antioxidant and cytoprotective function, the excretion of bilirubin in vivo is controlled by the UGT1A1 [5, 8]. Previous studies reported the circulating bilirubin levels were low in NAFLD or NASH patients, but some different phenomenons often were found. In addition, the relationship of UGT1A1 with developing risk of NAFLD was unknown. In this cross-sectional study, 125 patients with NAFLD and 47 healthy controls were recruited to investigate the role of circulating TB and UGT1A1 in NAFLD patients. As the results shown, there was no significant difference of circulating TB between NAFLD patients and controls. All the patients were subjected to the MRI-PDFF examination and divided into the mild steatosis, moderate steatosis, and severe steatosis groups according to the MRI-PDFF values. In this study, patients with moderate steatosis and severe steatosis had the higher circulating TB levels than controls, and not in the patients with mild steatosis. In addition, no significant difference of circulating UGT1A1 levels in the overall patients with NAFLD or each stage of steatosis between health controls.

Previous studies reported the association of circulating TB levels with the risk of NAFLD or NASH [10, 12, 20, 22, 23]. Whether the circulating bilirubin could act as the diagnostic biomarker for NAFLD attracted many attentions. Four studies in which patients with NAFLD were diagnosed by histologic NAS score tried to find the answer. Three studies of them found that unconjugated hyperbilirubinemia negatively associated with the liver damage in NAFLD patients in patients with NAFLD [20, 22, 23]. What’s more, another study found that circulating TB levels in adolescent NAFLD patients were negatively associated with the presence of NASH [10]. Nevertheless, Kunutsor et al. found that TB was not related to the developing risk of NAFLD which diagnosed by MR [12]. In this study, Spearman rank correlation coefficient analysis suggested that circulating TB was weakly associated with the liver fat fraction in patients with NAFLD. Logistic regression analysis suggested that circulating TB could increase the developing risk of NAFLD. These results were consistent with the higher circulating total bilirubin levels in patients with moderate and severe steatosis. When adjusted to the age, sex, and BMI, no significant association of circulating TB with the developing risk of NAFLD was observed, which suggested that circulating TB was not the independent risk factor for the risk of NAFLD. The inconsistent results of the association between circulating TB and the development of NAFLD may due to the different diagnstic methods of NAFLD and the complicated genetic background of subjects. More subjects diagnosed with biopsy should be recruited to illustrate the actual association of circulating TB with the risk of NAFLD in different district or countries.

UGT1A1 is not only an important enzyme in drug metabolism but also the sole enzyme that regulating the bilirubin metabolism [24, 25]. Expression of UGT1A1 is regulated by the combination of ubiquitous and developmental/tissue-specific transcription factors that act as sensors of substrate levels such as bile acids, steroid hormones, and xenobiotics. In addition, nonsynonymous changes in the exons and/or variations in regulatory regions could affect the expression and function of UGT1A1 [26]. Previous study reported that the UGT1A1*6 variant genotypes could decrease the risk of NAFLD, and the UGT1A1*28 variant genotypes did not correlate with the risk of NAFLD [14]. However, the relationship between circulating UGT1A1 levels and hepatic steatosis remains unclear. Several studies had demonstrated that the hepatic expressions of UGTs were altered in animals with a high-fat or high-sucrose diet [27, 28]. Furthermore, Hardwick et al. reported that the mRNA expression of UGT1A1 was raised during progressive stages of NAFLD patients [29]. This study explored the circulating UGT1A1 levels in patients with NAFLD for the first time. The mean circulating UGT1A1 levels in NAFLD patients were not differ to the controls. When the NAFLD patients were graded to mild, moderate, and severe steatosis by the MRI-PDFF values, the circulating UGT1A1 levels were positively correlated with liver fat fraction in patients with severe steatosis, which was consistent with the previous study [29]. In addition, the diagnostic value of circulating UGT1A1 or TB was assessed; the results suggested that the circulating UGT1A1 and TB did not possess the superior diagnostic accuracy for NAFLD.

The strength of this study was that all the subjects were diagnosed by MRI-PDFF, which can grade the steatosis into mild steatosis, moderate steatosis, and severe steatosis. There were several limitations in this study. Firstly, the number of subjects was relatively small, and the bias might be existed in the results. Secondly, all the patients with NAFLD were diagnosed by MRI-PDFF, although the diagnostic value of MRI-PDFF was high, the inconsistent results between this study and previous studies could be easier to explain if all the patients were subjected to liver biopsy. Thirdly, all the subjects were Chinese Han population, the results may be affected by the genetic and environmental factors. Fourthly, the data of direct bilirubin and indirect bilirubin of the subjects were not available in this study, the relationship between direct bilirubin and indirect bilirubin and UGT1A1 should be investigated in further study.

## Conclusions

In summary, this study investigated the circulating TB and UGT1A1 in the MRI-PDFF diagnosed NAFLD patients. The results indicated that circulating TB levels were significant high in patients with moderate steatosis and severe steatosis. In addition, the circulating TB was weak associated with liver fat fraction in patients with NAFLD. No significant difference of circulating UGT1A1 levels were observed between NAFLD patients and controls, but the circulating UGT1A1 levels were positively correlated with liver fat fraction in patients with severe steatosis. All the subjects were Chinese Han population, and the results indicated that circulating TB may associated with the severity of NAFLD and possess the potential of diagnostic biomarker for moderate and severe steatosis. When patients with NAFLD come out the increased serum TB and UGT1A1 levels, a severe steatosis of progression might be occurred, which can guide the clinical surveillance and treatment of patients with NAFLD. Further studies should be conducted in a large population in different regions to verify the relationship between NAFLD and circulating TB and UGT1A1.

## Data Availability

All data generated or analyzed during this study are included in this published article.

## Abbreviations

ALP: Alkaline phosphatase
ALT: Alanine aminotransferase
APOA1: Apolipoprotein A1
APOB: Apolipoprotein B
AST: Aspartate aminotransferase
BMI: Body mass index
FPG: Fasting plasma glucose
GGT: γ-Glutamyltransferase
HDL: High-density lipoprotein
LDL: Low-density lipoprotein
MRI-PDFF: Magnetic resonance imaging proton density fat fraction
NAFLD: Nonalcoholic fatty liver disease
NASH: Nonalcoholic steatohepatitis
SD: Standard deviation
TC: Total cholesterol
TG: Triglyceride
UA: Uric acid
UGT1A1: UDP-glucuronosyltransferases 1A1
